# Association between miRNAs expression and cognitive performances of Pediatric Multiple Sclerosis patients: A pilot study

**DOI:** 10.1002/brb3.1199

**Published:** 2019-01-17

**Authors:** Maria Liguori, Nicoletta Nuzziello, Marta Simone, Nicola Amoroso, Rosa Gemma Viterbo, Sabina Tangaro, Arianna Consiglio, Paola Giordano, Roberto Bellotti, Maria Trojano

**Affiliations:** ^1^ National Research Council Bari Unit Institute of Biomedical Technologies Bari Italy; ^2^ Unit for Severe Disabilities in Developmental Age and Young Adults, Developmental Neurology and Neurorehabilitation Scientific Institute IRCCS E. Medea Brindisi Italy; ^3^ Department of Basic Sciences, Neurosciences and Sense Organs University of Bari Bari Italy; ^4^ Dipartimento Interateneo di Fisica “M. Merlin” Università degli studi di Bari “A. Moro” Bari Italy; ^5^ Istituto Nazionale di Fisica Nucleare, Sezione di Bari Bari Italy; ^6^ General Paediatric Unit “B. Trambusti”, Azienda Policlinico‐Giovanni XXIII University of Bari Bari Italy

**Keywords:** bioinformatics, circulating biomarkers, cognitive dysfunctions, gene targets, HT‐NGS, miRNAs, molecular pathogenesis, MRI regional volumes, networks, pediatric multiple sclerosis

## Abstract

****Introduction**:**

The Pediatric onset of Multiple Sclerosis (PedMS) occurs in up to 10% of all cases. Cognitive impairment is one of the frequent symptoms, exerting severe impact in patients’ quality of life and school performances. The underlying pathogenic mechanisms are not fully understood, and molecular markers predictive of cognitive dysfunctions need to be identified. On these grounds, we searched for molecular signature/s (i.e., miRNAs and target genes) associated with cognitive impairment in a selected population of PedMS patients. Additionally, changes of their regional brain volumes associated with the miRNAs of interest were investigated.

**Methods:**

Nineteen PedMS subjects received a full cognitive evaluation; total RNA from peripheral blood samples was processed by next‐generation sequencing followed by a bioinformatics/biostatistics analysis.

**Results:**

The expression of 11 miRNAs significantly correlated with the scores obtained at different cognitive tests; among the others, eight miRNAs correlated with the Trail Making Tests. The computational target prediction identified 337 genes targeted by the miRNAs of interest; a tangled network of molecular connections was hypothesized, where genes like *BST1, NTNG2, SPTB, *and *STAB1*, already associated with cognitive dysfunctions, were nodes of the net. Furthermore, the expression of some miRNAs significantly correlated with cerebral volumes, for example, four miRNAs with the cerebellum cortex.

**Conclusions:**

As far as we know, this is the first evaluation exploring miRNAs in the cognitive performances of PedMS. Although none of these results survived the multiple tests’ corrections, we believe that they may represent a step forward the identification of biomarkers useful for monitoring and targeting the onset/progression of cognitive impairments in MS.

## 1 INTRODUCTION

Multiple sclerosis (MS) is a demyelinating autoimmune disease of the central nervous system (CNS) that usually affects young adults (Olsson, Barcellos, & Alfredsson, 2016), although the onset during childhood and adolescence is being increasingly recognized. Pediatric MS (PedMS) represents up to 10% of all MS cases (Banwell, 2014; Chitnis, Glanz, Jaffin, & Healy, 2009), the most prevalent course being the relapsing‐remitting (RR) with the higher relapse rate observed in the first 2 years of the disease course (Banwell, Ghezzi, Bar‐Or, Mikaeloff, & Tardieu, 2007; Waldman et al., 2016).

Among the first clinical evidences, the cognitive impairment is one of the most frequent in PedMS as in the adult form of the disease, exerting severe impact in patients’ quality of life and school performances. The pathogenic mechanisms underlying the neuropsychiatric and cognitive disorders in PedMS are not fully understood yet; they may be the result of the irreversible impact that inflammation (demyelination) and/or neurodegeneration produce on the ongoing maturation of the cognitive pathways (Amato et al., 2004, 2007; Di Filippo, Portaccio, Mancini, & Calabresi, 2018). This should be particularly plausible for the language skills that are more commonly involved in PedMS than in adult patients (Nunan‐Saah, Paulraj, Waubant, Krupp, & Gomez, 2015).

Advanced structural and functional MRI techniques provided interesting hypotheses about the mechanisms possibly implicated in the cognitive dysfunctions of PedMS. Among the other findings, structural damage to a set of posterior brain regions suggested that, through focal lesions and demyelinated plaques, degenerated axons may lead to deafferentation and atrophy, thus evoking a pivotal role of white matter (WM) distress, other than the gray matter (GM) loss, in the pathogenesis of the cognitive symptoms (Rocca et al., 2014).

On the other hand, molecular markers predictive of neuropsychological dysfunctions’ occurrence and progression still need to be identified in pediatric and in adult MS. microRNAs (miRNAs), a class of small noncoding RNA, seem to play a key role in complex diseases like MS as circulating regulatory factors that may serve, for example, as biomarkers of clinical activity (Baulina et al., 2018; Vistbakka, Sumelahti, Lehtimäki, Elovaara, & Hagman, 2018) or response to treatments (Fenoglio et al., 2016; Niwald, Migdalska‐Sęk, Brzeziańska‐Lasota, & Miller, 2017; Sáenz‐Cuesta et al., 2018). miRNA networks are actively involved in many neuropsychiatric disorder (Saab & Mansuy, 2014; Xu, Hsu, Karayiorgou, & Gogos, 2012), so the possibility that they may contribute to the pathogenic mechanisms underlying the cognitive disfunctions in MS is more than plausible. Furthermore, the identification of circulating miRNAs implicated in the occurrence of these disabling symptoms may be a valuable source of information also in the prospective of designating novel target for more efficient therapeutic efforts.

In order to investigate the role of miRNAs in the very early stages of MS, we performed an extensive analysis of miRNAs and mRNAs profiles derived from the peripheral blood samples of a selected PedMS population and a pediatric group of healthy subjects, and we identified 13 miRNAs and 4,306 mRNAs whose expression resulted significantly different between the two groups (Liguori, Nuzziello, Licciulli, et al., 2018b). No peculiar molecular signatures resulted associated with the PedMS clinical disability (EDSS) or cognitive dysfunctions, as simply categorized into cognitively impaired (CI) and preserved (CP) patients, according to international criteria (Amato et al., 2014).

However, given the very early assessment of their cognitive performances and since a detailed neuropsychological examination was available for these patients, we decided to look more deeply into this issue by searching for significant associations (if any) between the expressions of these miRNAs and the individual scores of the performed neuropsychological tests.

The main purpose of this analysis was to possibly identify those circulating miRNAs and their target genes that may be responsible for the pathogenic mechanism/s involved in the neuropsychological damage observed in PedMS, also providing a selection of circulating biomarkers (miRNAs) best suited for more target therapeutic efforts.

Given the availability of conventional MRI data, additional aim of this observation was to search for possible associations between the expression of the miRNAs of interest and the regional brain volumes of PedMS patients, following the intriguing hypothesis that miRNAs may also exert an impact in the genetic architecture of the neurodevelopment (Ziats & Rennert, 2014).

## 2 PATIENTS AND METHODS

This report is part of a multidisciplinary and longitudinal investigation performed on 19 Caucasian patients with PedMS (Banwell et al., 2007; Waubant et al., 2009) recruited within 5 years from the onset and followed up at the Department of Basic Sciences, Neurosciences and Sense Organs, University of Bari (Liguori, Nuzziello, Licciulli, et al., 2018b). Nine patients were under interferon β‐1a treatment at the time of the study entry; they all received neurological examination (EDSS; Kurtzke, 1983) and neuropsychological evaluation by using a battery of validated tests exploring the following cognitive domains (Amato et al., 2010):

1. Verbal learning and delayed recall through the Selective Reminding Test (SRT) and Selective Reminding Test—Delayed (SRT‐D) from Rao's battery.
2. Visuospatial learning through the Spatial Recall Test (SPART) and Spatial Recall Test—Delayed (SPART‐D) from Rao's battery.
3. Complex attention through the Symbol Digit Modalities Test (SDMT) from Rao's battery and the Trail Making Test (TMT‐A and TMT‐B).
4. Planning of executive functions through the Tower of London Test (TOL).
5. Expressive language through a semantic and phonemic verbal fluency test and an oral denomination test from the Aachener Aphasie Test.

Depression and fatigue were self‐assessed through the Children Depression Inventory (CDI) and the Fatigue Severity Scale (FSS), respectively. According to published guidelines, cognitive impairment (CI) was considered after a failure of at least three tests (Amato et al., 2010).

The study was approved by the Ethics Committee of Azienda Ospedaliera Policlinico, University of Bari. Since the study subjects were all under the age of 18 years, their legal tutors signed written informed consent forms (according to the Declaration of Helsinki) at the time of the enrollment.

### 2.1 Molecular analysis

Peripheral blood samples were collected from each PedMS patient at the study entry; total RNA isolation was followed by high‐throughput next‐generation sequencing (HT‐NGS) of both miRNAs and mRNAs compounds, and by qRT‐PCR validation only of the 13 miRNAs whose expressions resulted significantly different (DE) from those of a population of pediatric healthy controls (Liguori, Nuzziello, Licciulli, et al., 2018b).

Here, we used the expression data of the significant miRNAs (let‐7a‐5p, let‐7b‐5p, miR‐25‐3p, miR‐99b‐5p, miR‐125a‐5p, miR‐148b‐3p, miR‐181a‐5p, miR‐182‐5p, miR‐185‐5p, miR‐221‐3p, miR‐320a, miR‐652‐3p, and miR‐942‐5p), as resulted from the qRT‐PCR analysis according to the 2^−ΔΔCt^ method in a previous study (Liguori, Nuzziello, Licciulli, et al., 2018b).

### 2.2 Statistical analysis

Speaman rank‐order correlation test was applied in order to evaluate the following: (a) the correlations between the individual scores obtained during the neuropsychological (NPS) evaluation and the expression of each miRNA (fold change); (b) the correlation between the regional brain volumes and the miRNAs expressions; and (c) the correlations between the brain volumes and the NPS scores. Results were considered significant at *p* < 0.05. Adjustments for false discovery rate (FDR) for age at blood sample and gender were applied.

### 2.3 Target genes and pathway analysis

Starting from the results of the DE analysis performed on the two datasets (sRNAs and mRNAs)(Liguori, Nuzziello, Licciulli, et al., 2018b), the relationships between DE miRNAs and DE target genes were investigated through a bioinformatics approach. Their interactions were selected using two databases of experimentally validated bindings (miRTarBase and DIANA‐Tarbase). In order to consider the most reliable information about the interactions between the significant miRNAs and their target genes, we selected those bindings that were confirmed at least by the luciferase assay, among others reporter tests. Functional and pathway enrichment analysis of identified target DE genes was performed using the Database for Annotation, Visualization and Integrated Discovery (DAVID v6.8, http://david.abcc.ncifcrf.gov) tool. DAVID is a gene functional enrichment program that provides a large series of functional annotation tools and pathway databases (e.g., KEGG, BioCarta, Reactome databases). The statistical significance was determined using the one‐tailed Fisher's exact test followed by the Benjamini correction; adjusted *p*‐value <0.05 was set as the threshold value. Details of methods and software used for the analysis have been already published (Liguori, Nuzziello, Introna, et al., 2018a; Liguori, Nuzziello, Licciulli, et al., 2018b).

### 2.4 Regional MRI

MRI scan was acquired at the study entry (T0) in all the 19 PedMS; however, only the images of 12 of them were available for this analysis. MRI examinations were performed with a 1,5 Tesla GE Signa MR System. Conventional sequences, consisting of dual‐echo, in order to obtain a T2‐weighted image, and fluid‐attenuated inversion recovery (FLAIR), were acquired in axial and coronal orientation covering the whole brain. Double fast spin‐echo sequence (TR/TE1/TE2, 2,100 = 12 = 96 ms; matrix, 345,512) and the FLAIR sequence (TR/TE, 8,152 = 102 ms; matrix, 256,256) were performed with 20 slices, 5 mm thickness, 1:0 mm interslice gap, and 24 cm FOV. MRI data were processed with the publicly available brain segmentation tool FreeSurfer v.5.1 in order to obtain a 181‐feature representation. The processing pipeline provides, for each subject, GM and WM volumes of subcortical brain structures along with the intracranial volume in mm^3^; in addition, FreeSurfer yields the average thickness of specific cerebral regions. Data were initially cleaned by removing constant features; the remaining data (a matrix with 12 subjects and 150 features) were standardized in order to have null average and unitary variance.

## 3 RESULTS

The main demographic and clinical characteristics of the PedMS population are summarized in Table 1 and detailed in Liguori, Nuzziello, Licciulli, et al. (2018b). Seven PedMS patients failed at least three tests so were classified as CI; 11 subjects were described as CP; one patient refused to be tested. No significant differences were found between the clinical and demographic features of CP and CI PedMS patients (*p* > 0.05), and no molecular profiles resulted associated with these two phenotypes (Liguori, Nuzziello, Licciulli, et al., 2018b).

**Table 1:** Demographic and clinical features of the PedMS population

	PedMS (19)
Age at onset (years, mean ± *SD*)	12.6 ± 3.2
Female/male	10/9
Age (years, mean ± *SD*)	15.5 ± 2.7
Disease duration (years, mean ± *SD*)	2.8 ± 3.3
Disease course	RR
EDSS (median, range)	3 (1.5–6)
Disease‐modifying treatment (yes/no)	9/10
Education (years, mean ± *SD*)	9.6 ± 2.5
Verbal memory	
SRT‐LTS (mean ± *SD*)	35.4 ± 14.8
SRT‐CLTR (mean ± *SD*)	27.2 ± 15.7
SRT‐D (median, range)	7.5 (4–12)
Visual‐spatial memory	
SPART (mean ± *SD*)	21.7 ± 4.9
SPART‐D (median, range)	8 (4–10)
Attention and IPS	
SDMT (mean ± *SD*)	41.8 ± 12.8
TMT‐A (mean ± *SD*)	41.4 ± 13.7
TMT‐B (median, range)	77.5 (38–151)
Executive functioning	
TOL (mean ± *SD*)	28 ± 4.8
Expressive language	
SVFT (mean ± *SD*)	21.1 ± 11.9
PVFT (mean ± *SD*)	16.2 ± 7.7

LTS, long term storage; CTRL, consistent long term retrieval; SVFT, semantic verbal fluency test; PVFT, phonemic verbal fluency test.

### 3.1 miRNas and cognitive functions

The expression of 11/13 miRNAs correlated with the scores obtained in several neuropsychological tests (Table 2). Among the others, a panel composed of eight miRNAs inversely correlated with the scores obtained at the TMT‐A and TMT‐B tests (*p* < 0.05). The verbal memory performances positively correlated with the expressions of miR‐182‐5p and miR‐942‐5p. The TOL test was significantly associated with the expressions of miR‐125a‐5p and miR‐221‐3p, whereas, in the domain of the expressive language, the SVFT test correlated significantly with miR‐181a‐5p. Finally, the higher expressions of miR‐320a correlated with the higher depression and the fatigue scores, and the latter also correlated with the expression of miR‐99b‐5p. None of these correlations remained significant after FDR corrections.

**Table 2:** Correlations between miRNAs expressions and the NPS scores

	Verbal memory			Attention and IPS		Executive functions	Expressive language	Depression	Fatigue
	SRT_LTS p‐values (*r*_s_)	SRT_CLTR *p*‐values (*r*_s_)	SRT_D *p*‐values (*r*_s_)	TMT_A *p*‐values (*r*_s_)	TMT_B *p*‐values (*r*_s_)	TOL *p*‐values (*r*_s_)	SVFT *p*‐values (*r*_s_)	CDI *p*‐values (*r*_s_)	FSS *p*‐values (*r*_s_)
miR25_3p					0.027 (−0.52)				
miR125a_5p				0.013 (−0.57)	0.007 (−0.61)	0.048 (0.47)			
miR942_5p		0.05 (0.47)			0.032 (−0.51)				
miR221_3p						0.012 (−0.58)			
miR652_3p				0.021 (−0.54)					
miR182_5p	0.023 (0.53)	0.004 (0.64)	0.02 (0.54)						
miR185_5p				0.009 (−0.6)					
miR181a_5p							0.036 (0.49)		
miR320a					0.018 (−0.56)			0.038 (0–49)	0.039 (0.49)
miR99b_5p					0.044 (−0.48)				0.038 (0.49)
miR148b_3p				0.49 (−0.47)	0.012 (−0.58)				

Note

Speaman rank‐order correlation tests: *p* < 0.05.

### 3.2 miRNAs/mRNAs integrated analysis in cognitive performances

**Figure 1:**
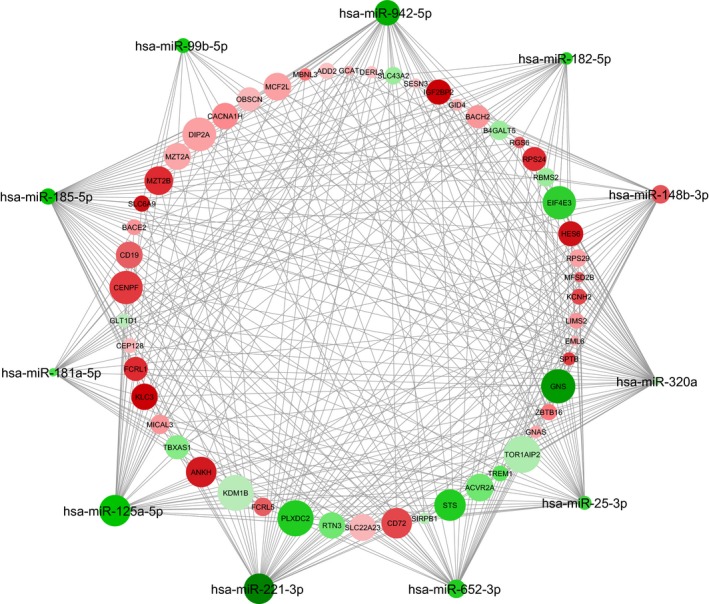
Hypothesized molecular network of cognitive abilities in PedMS. By using the connections between the first genes targeted by at least 6 out of the 11 miRNAs significantly associated with the NPS scores, the following network was composed (Cytoscape software 3.5.1). The node intensity color is proportional to the fold change values (red: under‐regulated; green: up‐regulated); the node size is proportional to the number of miRNA/mRNA connections. Please note that, for this representation, only down‐regulated genes were selected as target of up‐regulated miRNAs and *vice versa *for down‐regulated miRNAs

The computational analysis of the target predictions identified 337 DE genes targeted by the 11 miRNAs that significantly correlated with the NPS scores in our PedMS population. By looking at the top shared (at least by six miRNAs), a tangled network of molecular connections possibly underlying the cognitive abilities was built (Figure 1). As nodes of the net, *BRI3, SPTB, CACNA1H, DIP2A, NTNg2, RGS6, IGF2BP2, KCNH2, RTN3, RAB11FIP5, and STAB1* have been already reported associated with cognitive functions (Table 3, see the Discussion for comments). None of these correlations remained significant after FDR corrections.

**Table 3:** The most significant miRNA's predicted targets (either up‐ and down‐regulated in our analysis)

Predicted target genes	Associated diseases with cognitive impairment	Number of shared miRNAs
*BRI3*	AD	10
*BST1*	Cognition in PD, autism	10
*RGS6*	AD	9
*CD72*	MS	8
*DIP2A*	AD, dyslexia, autism	8
*FCRL1*	MS, PD	8
*NTNG2*	Amygdala/cognition, bipolar disorder	8
*RAB11FIP5*	Depression	8
*SIRPB1*	Impulsive behavior, AD	8
*SPTB*	Cognitive processes	8
*ZBTB16*	AD, depression	8
*BACE2*	AD, neurodegenerative disorders	7
*BACH2*	MS	7
*CACNA1H*	MS	7
*IGF2BP2*	AD, PD	7
*RTN3*	AD	7
*SESN3*	Aging and degeneration	7
*TBXAS1*	MS, schizophrenia	7
*WARS*	Intellectual disability	7
*ADD2*	Schizophrenia	6
*FAM46A*	AD, depression	6
*FCRL5*	MS, AD	6
*FKBP1A*	Depression and response to therapy	6
*GNAS*	Animal cognition	6
*HES6*	Mood disorder	6
*KCNH2*	Schizophrenia	6
*MCF2L*	AD	6
*SHTN1*	MS	6
*SLC27A3*	Autism	6
*SPI1*	PD, AD	6
*STAB1*	Bipolar disorder	6
*STS*	ADHD	6
*TREM1*	AD	6

Note

AD, Alzheimer's disease; ADHD, attention‐deficit and hyperactive disorder; MS, multiple sclerosis; PD, Parkinson's disease.

### 3.3 miRNAs and regional volumes

The correlation analysis between brain volumes (as they were generated by the FreeSurfer pipeline) and the miRNAs expressions revealed several significant correlations (Table 4). Among the others, the expression of four miRNAs (miR‐25‐3p, miR‐125a‐5p, miR‐221‐3p, and miR‐320a) was significantly associated with total cerebellum cortex, whereas the total hippocampus volume correlated with miR‐182‐5p expression. None of these correlations remained significant after FDR corrections.

**Table 4:** Correlations between miRNAs expressions and regional brain volumes

	Cerebellum cortex *p*‐values (*r*_s_)	Hippocampus *p*‐values (*r*_s_)	Corpus callosum *p*‐values (*r*_s_)	R‐Frontal Lobe *p*‐values (*r*_s_)	R‐Parietal Lobe *p*‐values (*r*_s_)	L‐Temporal Lobe *p*‐values (*r*_s_)	L‐Occipital Lobe *p*‐values (*r*_s_)	R‐Occipital Lobe *p*‐values (*r*_s_)	L‐Cingulate *p*‐values (*r*_s_)	R‐Cingulate *p*‐values (*r*_s_)
miR‐25‐3p	0.001 (−0.82)									
miR‐125a‐5p	0.036 (−0.61)								0.042 (0.59)	
miR‐221‐3p	0.042 (0.59)									
miR‐652‐3p						0.042 (0.59)				
miR‐182‐5p		0.033 (−0.62)			0.042 (−0.59)					
miR‐320a	0.02 (−0.66)				0.017 (−0.67)			0.033 (−0.62)		
miR‐148b‐3p								0.026 (−0.64)		0.008 (−0.72)

Note

Speaman rank‐order correlation tests: *p* < 0.05.

### 3.4 Pathway analysis

**Figure 2:**
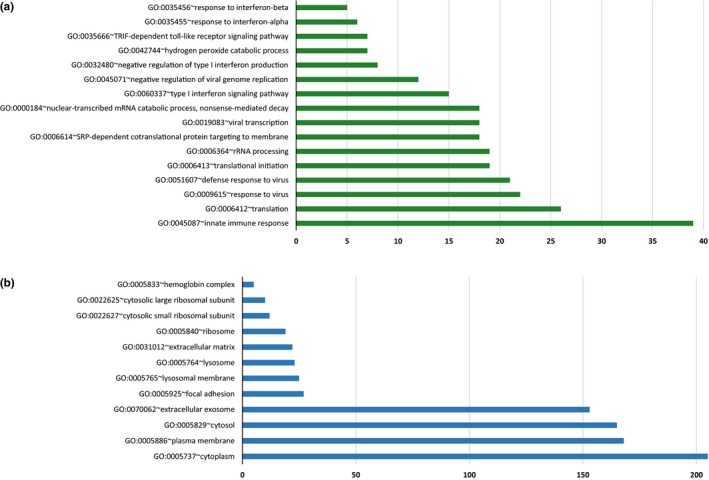
Frequencies of GO terms evoked by the significant target genes possibly implicated in PedMS cognitive dysfunctions. Histogram illustrates the GO terms (a: biological processes; b: cellular components**)** associated with assembled subnetworks (DAVID software v6.8). Please note that this analysis was performed by using all the predicted target genes (computationally or experimentally validated, see Methods for details) of the significant 11 miRNAs correlating with the score of the different cognitive performances (either up‐ or down‐regulated genes)

The most representative molecular functions (GO terms) was the protein binding (adjusted *p*‐value = 1.01e10^−05^); the analysis of the biological processes revealed that the significant genes were mostly involved in innate immune responses (adjusted *p*‐value = 4.9e10^−08^), translation (adjusted *p*‐value = 1.4e10^−06^), response and defense response to virus (adjusted *p*‐value = 8.2e10^−11^ and 6.5e10^−07^, respectively) (Figure 2a). The most represented genes of cellular components were identified within the cytoplasm (adjusted *p*‐value = 0.00125) (Figure 2b).

### 3.5 Cognitive functions and regional volumes

Regional cerebral volumes significantly correlated with specific cognitive functions. In details, the volumes of the corpus callosum significantly correlated with the scores obtained at the TMT‐A (*r*_s_ = −0.64, *p* = 0.025), SPART‐D (*r*_s_ = 0.68, *p* = 0.015), and CDI (*r*_s_ = −0.6, *p* = 0.04) tests; the left temporal lobe significantly correlated with the scores obtained at the SRT‐LTS (*r*_s_ = 0.86, *p* = 0.0001), SRT‐CLTR (*r*_s_ = 0.75, *p* = 0.005), SDMT (*r*_s_ = 0.6, *p* = 0.04), SPART‐D (*r*_s_ = 0.6, *p* = 0.04), TOL (*r*_s_ = 0.63, *p* = 0.03), and CDI (*r*_s_ = −0.66, *p* = 0.02). Further significant correlations were the following: the cerebellum white matter with the scores obtained at the TOL test (*r*_s_ = −0.78, *p* = 0.003); the left frontal lobe with the SRT‐D (*r*_s_ = −0.73, *p* = 0.007); and the right occipital lobe with the SDMT test (*r*_s_ = 0.65, *p* = 0.021).

## 4 DISCUSSION

Children and adolescents diagnosed with MS are particularly vulnerable to cognitive dysfunctions since the disease occurs during the growing phases of brain development, myelination, and maturation of the neural network. On the other hand, it has been recognized that growing brain still holds a basic reorganization until the adolescence, mostly due to elimination of synapses in the cortical circuits, increase in white matter, and changes in neurotransmitter systems, suggesting that an open window for peculiar changes in cognitive functions does exist both in healthy and in specific pathological conditions (Konrad, Firk, & Uhlhaas, 2013).

In this report, by looking into the expression of 13 miRNAs that characterized a small but homogeneous group of PedMS patients (Liguori, Nuzziello, Licciulli, et al., 2018b), we narrowed down 11 miRNAs (miR‐25‐3p, miR‐99b‐5p, miR‐125a‐5p, miR‐148b‐3p, miR‐181a‐5p, miR‐182‐5p, miR‐185‐5p, miR‐221‐3p, miR‐320a, miR‐652‐3p, and miR‐942‐5p) that resulted significantly associated with different cognitive abilities. Of interest, eight miRNAs were found implicated in the domains of attention and information processing speed, frequent cognitive dysfunctions in PedMS that exert a significant impact in patients’ life and school performances (Ghezzi, Goretti, Portaccio, Roscio, & Amato, 2010; Goretti et al., 2012; Julian et al., 2013). Some of the 11 miRNAs have been already reported associated with cognitive abilities in vitro or in vivo evaluations (Dutta et al., 2013; Kos et al., 2016; Olde Loohuis et al., 2012; Woldemichael & Mansuy, 2016; Zhang, Chen, Zhang, & Xu, 2017), whereas for others, the association is novel so far. Above all, in our view this preliminary evidence may represent a step forward the identification of molecular markers of cognitive impairment to monitor during the MS course, and it may help to hypothesize more focused pharmacological and rehabilitative strategies.

Mature miRNAs bind the target sites of protein‐coding genes that lead to the repression of their translation; in mammals, they are predicted to exert post‐transcriptional control on over 60% of the protein‐coding genes (Keller et al., 2011). A deregulation of miRNA expression seems to be involved in a broad spectrum of cellular and biological processes including developmental timing, hematopoiesis, organogenesis, apoptosis, and cell proliferation (Soltanzadeh‐Yamchi, Shahbazi, Aslani, & Mohammadnia‐Afrouzi, 2018). On these grounds, it is plausible that miRNAs networks may also impact the human brain development, leading to possible significant changes in regional cerebral volumes with/out subsequent functional (i.e., cognitive) consequences (Xue, Zhuo, & Shan, 2017).

Following this suggestion, a quite recent study analyzed the differential expression of miRNome in 82 apparently normal postmortem human brain tissue samples belonging to 18 individual donors and spanning through 19 years of age. By looking at different areas, the study showed differentially expressed miRNAs and miRNAs patterns characterizing the cerebral areas, the cerebellum being the most influenced area compared to the other regions in all the studied intervals (65–252 DE miRNAs; Ziats & Rennert, 2014). On the other hands, peculiar miRNA signatures seem to characterize the individual lesion load and brain atrophy of MS patients; although none of the identified miRNAs associated with the MRI outcomes survived from the correction for multiple comparisons, they pointed the attention to some miRNAs (miR‐92a‐3p, miR‐142‐5p, miR‐143‐3p, miR‐181c‐3p, miR‐181c‐5p, miR‐375, miR‐486‐5p, and miR‐629‐5p) as candidate surrogate markers for the MS monitoring (Regev et al., 2017). So far, no evidences have been reported about correlations between miRNAs expressions and in vivo regional brain volumes during MS.

In the present observation, we did identify several significant associations between the expressions of several miRNAs and the volume of few brain regions. With caution due to the limited number of MRI examinations (i.e., we were able to analyze only 12 scans), the most interesting results pointed on the cerebellum cortex, which volume significantly correlated with the expression of four miRNAs (miR‐25‐3p, miR‐125a‐5p, miR‐221‐3p, and miR‐320a), in our view confirming the complex molecular influence of this region compared to other areas (Ziats & Rennert, 2014). Furthermore, lower volumes of the hippocampus correlated with the higher expression of miR‐182‐5p, already reported dysregulated in the cortex and hippocampus of the animal model of prion disease (Boese et al., 2016). No gender‐related molecular differences with brain regions were found in the examined PedMS population. A larger sample size will possibly add more information on this interesting issue, together with the possibility to compare the MRI data with a population of age‐matched healthy controls (not allowed for the present investigation by the local Ethics Committee).

The same limitation of sizing concerns the correlations between the regional MRI volumes and the cognitive performances. However, it is worthy to note that most of our significant results were confirmations of published reports. Among the others, we obtained significant correlations between the overall corpus callosum atrophy and both the executive functions and depression (Benedict, Ramasamy, Munschauer, Weinstock‐Guttman, & Zivadinov, 2009; Johnson‐Markve, Lee, Loring, & Viner, 2011; van Schependom & Nagels, 2017), whereas the right occipital lobe volume was found significantly related to the performances obtained at SDMT (Akbar et al., 2016).

Finally, the computational analysis identified several genes that represent the target of most of the significant miRNAs (see Table 4). Of interest, some of these genes have been reported implicated in diseases with cognitive impairment, for example, *BRI3, RGS6, DIP2A, ZBTB16, BACE2, SIRBP1, IGF2BP2, FCRL5, RTN3, FAM46A, MCF2L, SPI1,* and *TREM1* in Alzheimer's disease (Abd‐Elrahman, Hamilton, Vasefi, & Ferguson, 2018; Chung et al., 2015; Comabella et al., 2016; Dashinimaev, Artyuhov, Bolshakov, Vorotelyak, & Vasiliev, 2017; De Jager et al., 2014; Gaikwad et al., 2009; Gasparoni et al., 2018; Matsuda, Matsuda, & D'Adamio, 2009; Moon et al., 2015; Replogle et al., 2015; Schott et al., 2016; Shi, Ge, He, Hu, & Yan, 2017); or in clinical conditions characterized by behavioral changes, such as *NTNG2* in cognitive abnormalities associated with defective axonal amygdalar projections (Huang et al., 2014) or bipolar disorders (Egger et al., 2014), or *RAB11FIP5, WARS, and HES6* in depression and other mood disorders (Bacaj, Ahmad, Jurado, Malenka, & Sudhof, 2015; Glubb, Joyce, & Kennedy, 2009; Musante et al., 2017). Furthermore, the experimental ablation of *CACNA1H*, a gene already associated with the RR course of MS (Sadovnick et al., 2017), was able to trigger affective disorders including anxiety and hippocampus‐dependent recognition memories (Gangarossa, Laffray, Bourinet, & Valjent, 2014).

In vitro validation of the significant target genes will be the next following step of our investigation. In fact, we believe that looking more deeply in this tangled network might represent a valuable strategy for enlightening the molecular background of cognitive dysfunctions in MS, and it would be interesting to evaluate other cognitive diseases of the neurodevelopment in order to investigate the molecular cross talk, if any, between the pathological networks of cognition.

In conclusion, as far as we know, this is the first evaluation exploring the association of miRNAs’ expressions with the cognitive performances of PedMS patients. Although the reported results did not survive the corrections for multiple tests possibly due to the small number of cases, in our view some associations deserve further investigation. Furthermore, the integrated miRNAs/mRNAs analysis enabled us to draw an interesting network of molecular connections, thus enlightening some genes that seem to play the role of functional hubs. If confirmed in larger PedMS populations and compared to a population of adult MS patients, this would be a first step toward the development of individualized therapies targeting the cognitive dysfunctions, as well as for the selection of circulating biomarkers (miRNAs) for monitoring the onset and progression of the neuropsychological changes during the course of the disease.

## CONFLICT OF INTEREST

None declared.
